# Publisher Correction: SEL1L-HRD1 interaction is required to form a functional HRD1 ERAD complex

**DOI:** 10.1038/s41467-024-47251-2

**Published:** 2024-04-15

**Authors:** Liangguang Leo Lin, Huilun Helen Wang, Brent Pederson, Xiaoqiong Wei, Mauricio Torres, You Lu, Zexin Jason Li, Xiaodan Liu, Hancheng Mao, Hui Wang, Linyao Elina Zhou, Zhen Zhao, Shengyi Sun, Ling Qi

**Affiliations:** 1 Department of Molecular Physiology and Biological Physics, University of Virginia, School of Medicine, Charlottesville, VA 22903 USA; 2 Department of Molecular & Integrative Physiology, University of Michigan Medical School, Ann Arbor, MI 48105 USA; 3 Zilkha Neurogenetic Institute, Keck School of Medicine of University of Southern California, Los Angeles, CA 90033 USA; 4 Department of Pharmacology, University of Virginia, School of Medicine, Charlottesville, VA 22908 USA; 5 Present Address: Life Sciences Institute and Department of Cell & Developmental Biology, University of Michigan Medical School, Ann Arbor, MI 48109 USA; 6 Present Address: Department of Radiology and Biomedical Imaging, University of California San Francisco, San Francisco, CA 94143 USA

**Keywords:** Endoplasmic reticulum, Ubiquitin ligases, ER-associated degradation

Correction to: *Nature Communications* 10.1038/s41467-024-45633-0, published online 16 February 2024

The original version of this Article omitted a reference to previous work in ‘Wei, X., Lu, Y., Lin, L.L. et al. Proteomic screens of SEL1L-HRD1 ER-associated degradation substrates reveal its role in glycosylphosphatidylinositol-anchored protein biogenesis. Nat Commun 15, 659 (2024). 10.1038/s41467-024-44948-2’. This has been added as reference 85 in the first sentence of the Methods subsection ‘Mass spectrometry’: ‘The SEl1L-IP in HEK293T cells has been described previously^85^.’ The reference number replaces the placeholder text ‘(please insert PMID:38253565)’ which was inadvertently included previously. This has been corrected in both the PDF and HTML versions of the Article.

