# Supplementary material for: SEL1L-HRD1 interaction is required to form a functional HRD1 ERAD complex
Source: Nat Commun. 2024 Feb 16;15:1440. doi: 10.1038/s41467-024-45633-0 (PMC10873344; doi:10.1038/s41467-024-45633-0)
Supplement: Supplementary file 1 — Supplementary Information [file 41467_2024_45633_MOESM1_ESM.pdf]

Supplementary Information

SEL1L-HRD1 interaction is required to form a functional HRD1 ERAD complex

Liangguang Leo Lin[1], Huilun Helen Wang[1], Brent Pederson[2], Xiaoqiong Wei[1], Mauricio Torres[2], You Lu[2,5], Zexin Jason Li[1], Xiaodan Liu[3,6], Hancheng Mao[2], Hui Wang[1], Linyao Elina Zhou[1], Zhen Zhao[3], Shengyi Sun[4]*, Ling Qi[1]*

* Correspondence: xvr2hm@virginia.edu (L.Q.); bjk5fz@virginia.edu (S.S.)

This PDF file includes:

Supplementary Fig. 1-8
Supplementary Table 1 and 2

Supplementary Figures and Legends

a

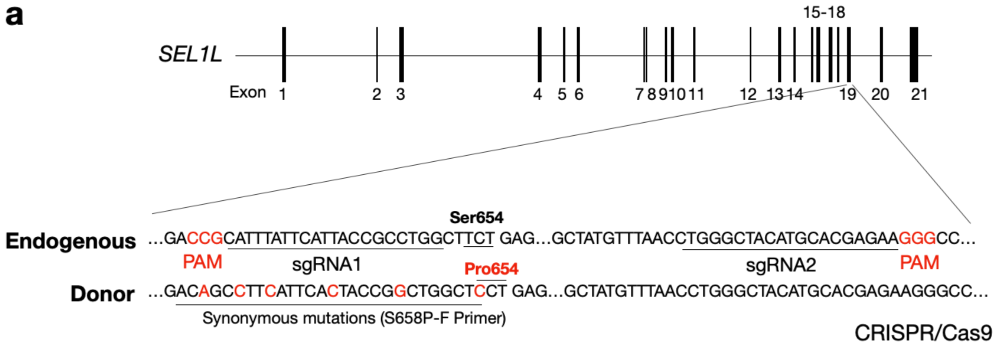

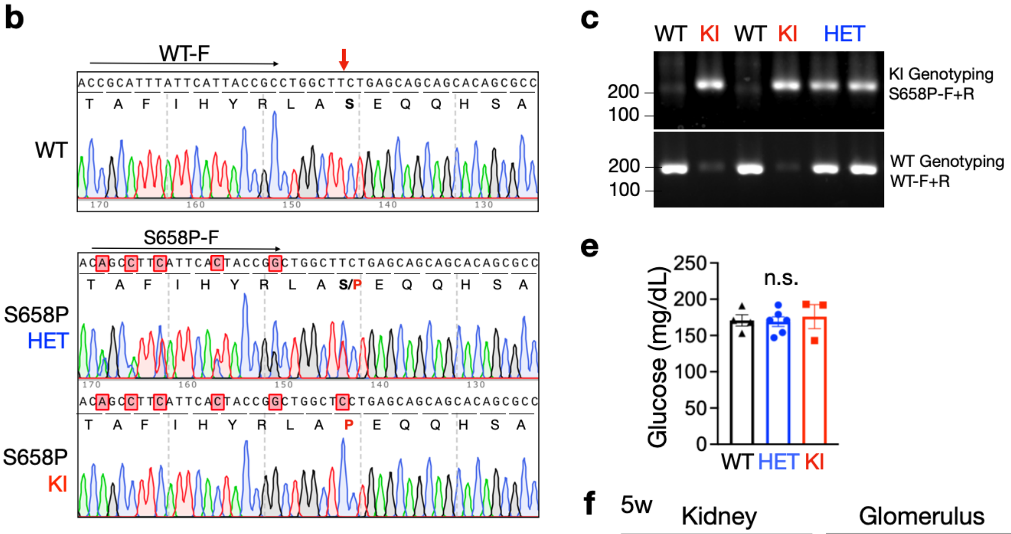

d

Embryonic days (E)	Total	WT	HET	KI
Expected ratio		25%	50%	25%
E10.5-E12.5	129	32 (24.8%)	75 (58.1%)	22 (17.1%)
E14.5-E16.5	116	33 (28.4%)	67 (57.8%)	16 (13.8%)
Post-natal day 21	247	75 (30.4%)	145 (58.7%)	27 (10.9%)

f

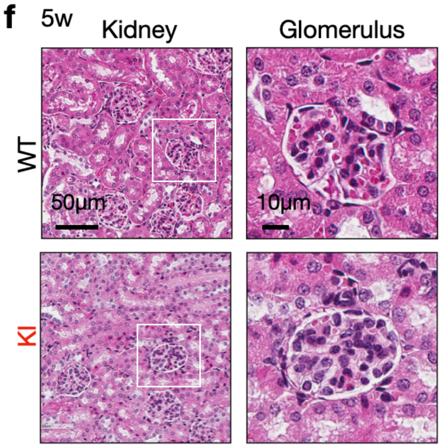

Supplementary Fig. 1. Generation of *SEL1L*^{S658P} KI mice.
(a) Schematic diagram of mouse *Sel1L* gene, with sequences of the CRISPR target loci and donor DNA template illustrated. SEL1L S654 in mice is homologous to S658 in human, thus the KI mouse model is referred to as *SEL1L*^{S658P} throughout the paper for simplicity. **(b)** Sequence chromatograms for the target site with nucleotide change highlighted in red arrow, and synonymous mutations highlighted in red shaded box. Forward primers used for genotyping are illustrated. **(c)** Representative genotyping results of mice from two founders. **(d)** Timed pregnancy and frequency of different genotypes showing partial embryonic lethality of the KI mice. **(e)** Ad libitum blood glucose levels of 5-week-old mice (n = 4, 6 and 3 mice for WT, HET and KI). **(f)** Hematoxylin & eosin (H&E) stained sections of kidneys and glomeruli from 5-week-old mice (n = 3 mice per group).

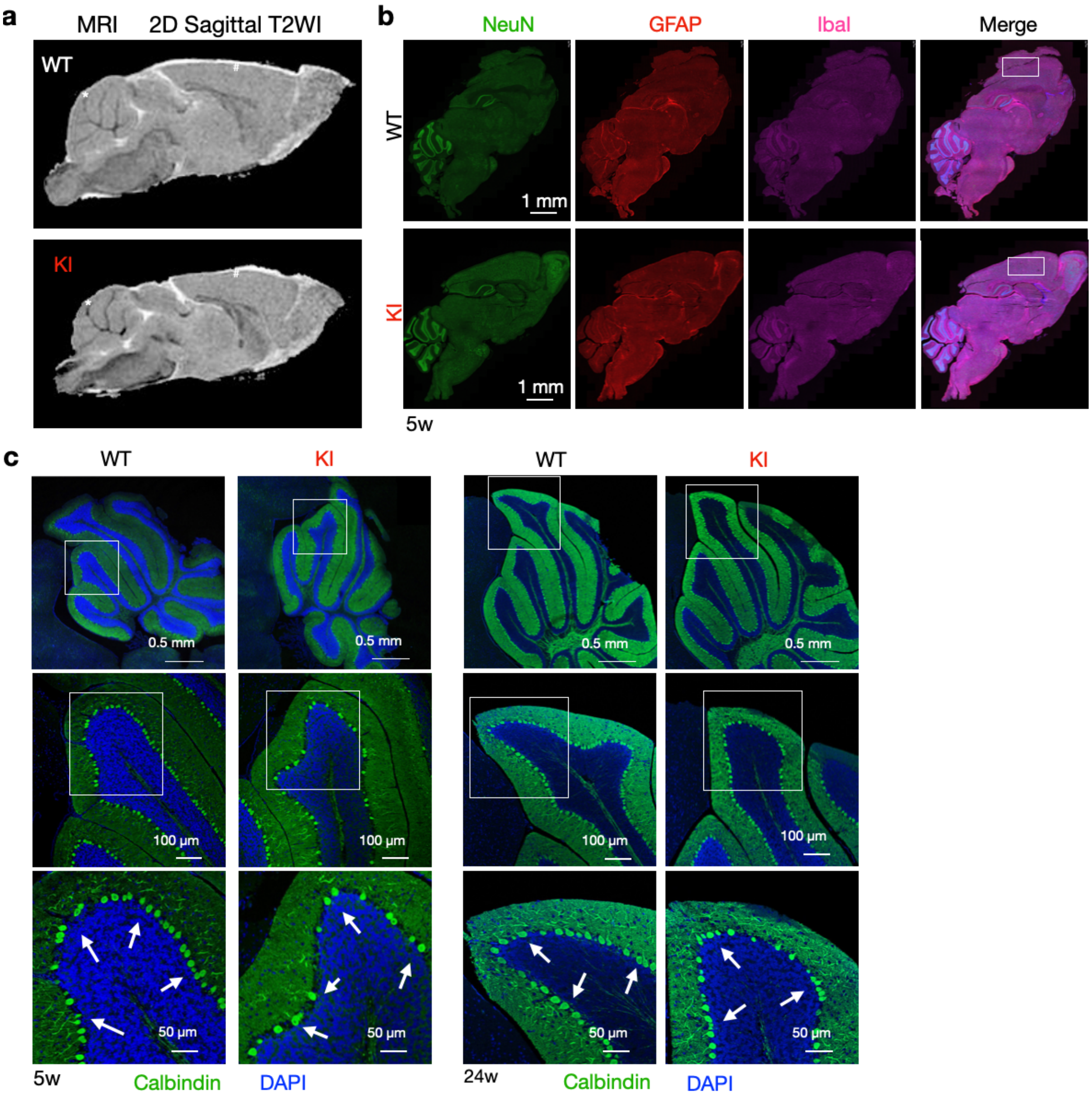

Supplementary Fig. 2. MRI and immunofluorescence analyses of brains and cerebellums from *SEL1L*^{S658P} KI and WT littermates.

(a) Morphological sagittal T2-weighted images (T2WI) of 5-week-old mouse brains using MRI analysis (n = 5 mice per group). **(b)** Representative confocal images of NeuN (green), GFAP (red), and Ibal (purple) staining for neurons, astrocytes, and microglia, respectively, in the brains of 5-week-old mice (n = 2 mice per group). Boxed areas are shown in Fig. 3e. **(c)** Representative confocal images of Calbindin (green) staining in the cerebellum of 5- and 24-week-old mice. White arrows, Purkinje cells (n = 3 mice per group).

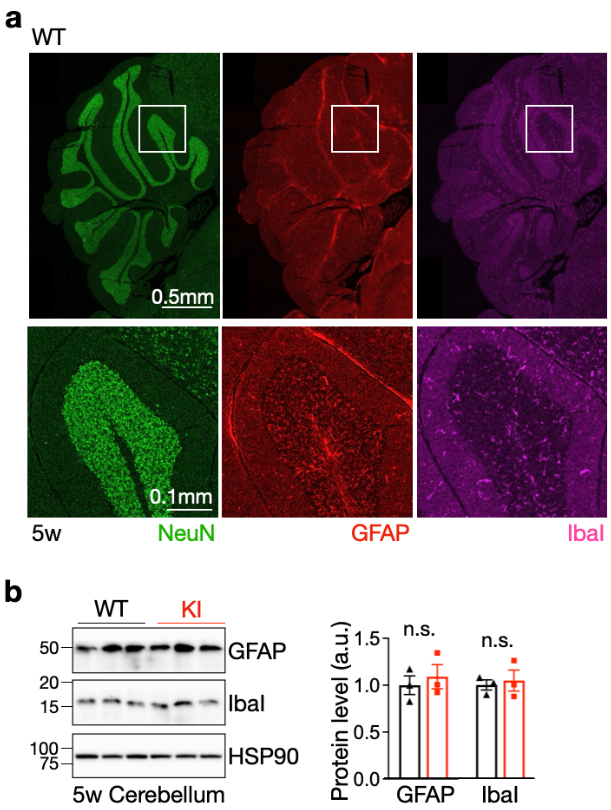

Supplementary Fig. 3. Immunofluorescence and Western blot analyses of cerebellum in *SEL1L*^{S658P} KI and WT littermates.

(a) Representative confocal images of NeuN (green), GFAP (red), and IbaI (purple) staining for neurons, astrocytes, and microglia, respectively, in the cerebellum of 5-week-old mice (n = 2 mice per group). **(b)** Western blot analysis of GFAP and IbaI in the cerebellum of 5-week-old mice with quantitation shown on the right (n = 3 mice per group). Values, mean ± SEM. n.s., not significant by two-tailed Student's *t*-test.

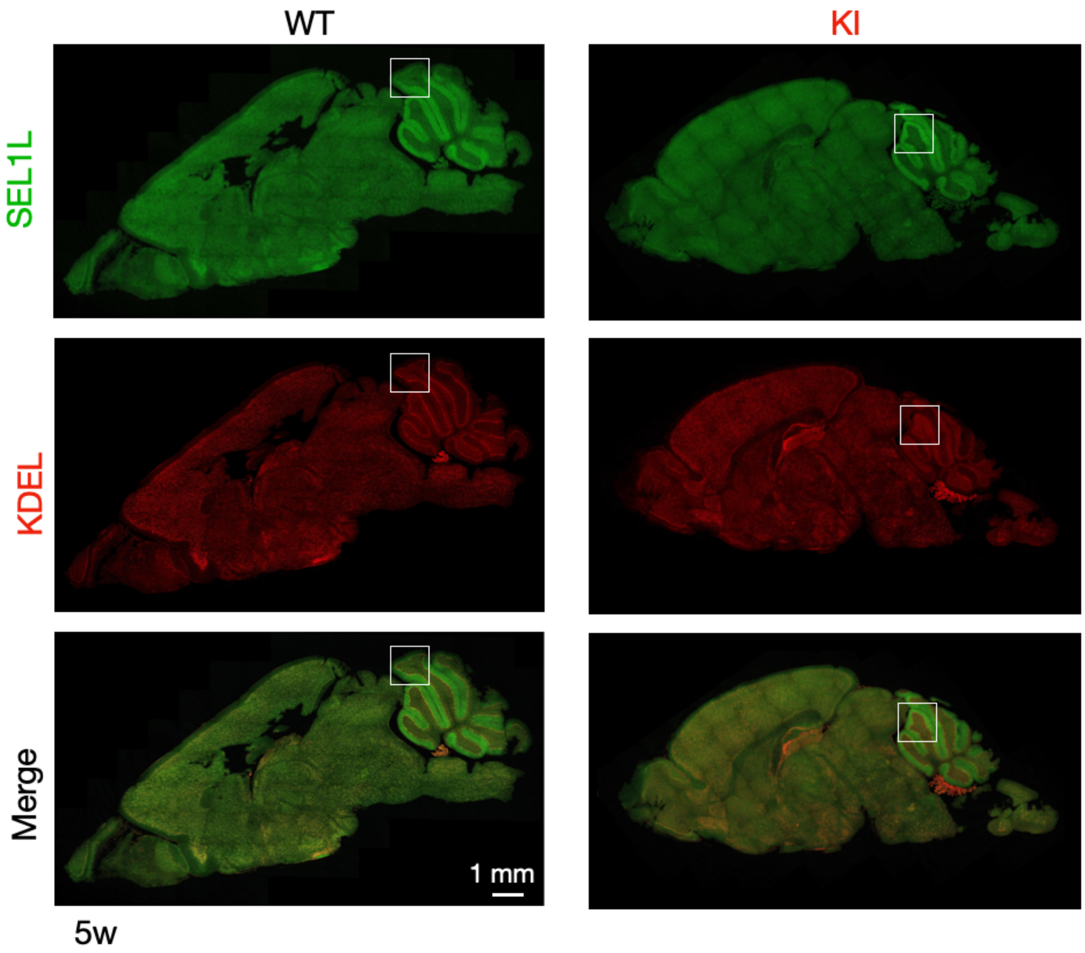

Supplementary Fig. 4. SEL1L is highly expressed in the cerebellum. Representative confocal images of SEL1L (green) and KDEL (ER marker, red) in the whole brains of 5-week-old mice (n = 2 mice per group). Boxed areas are shown in Fig. 5a.

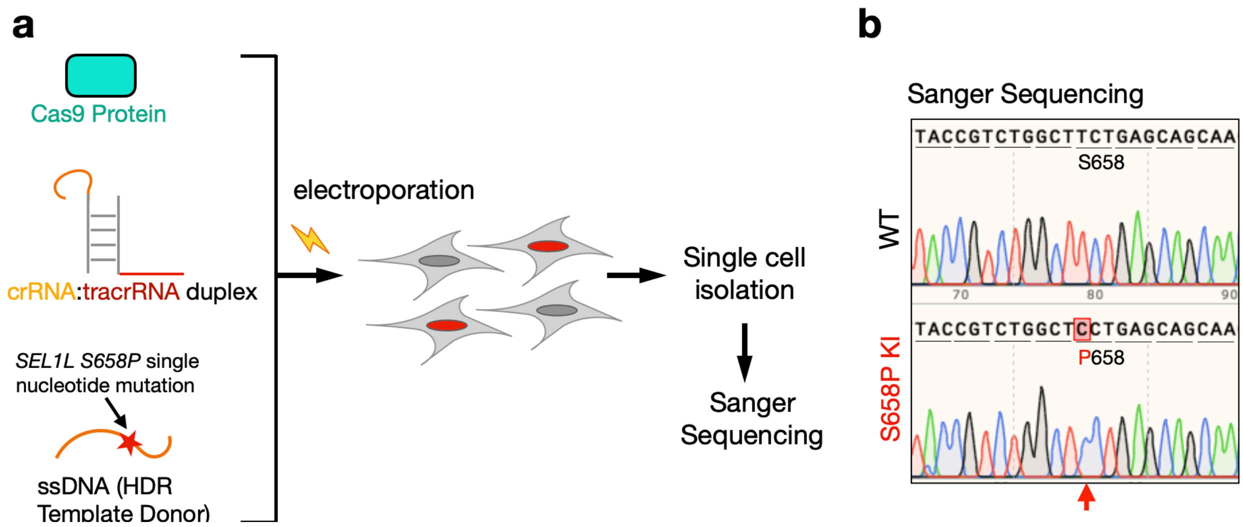

Supplementary Fig. 5. Generation of *SEL1L*^{S658P} KI HEK293T cells.
(a) Schematic diagram of the generation of *SEL1L*^{S658P} KI HEK293T using the CRISPR/Cas9 technology and **(b)** confirmation by Sanger sequencing. The shaded red box and arrow indicated the mutation.

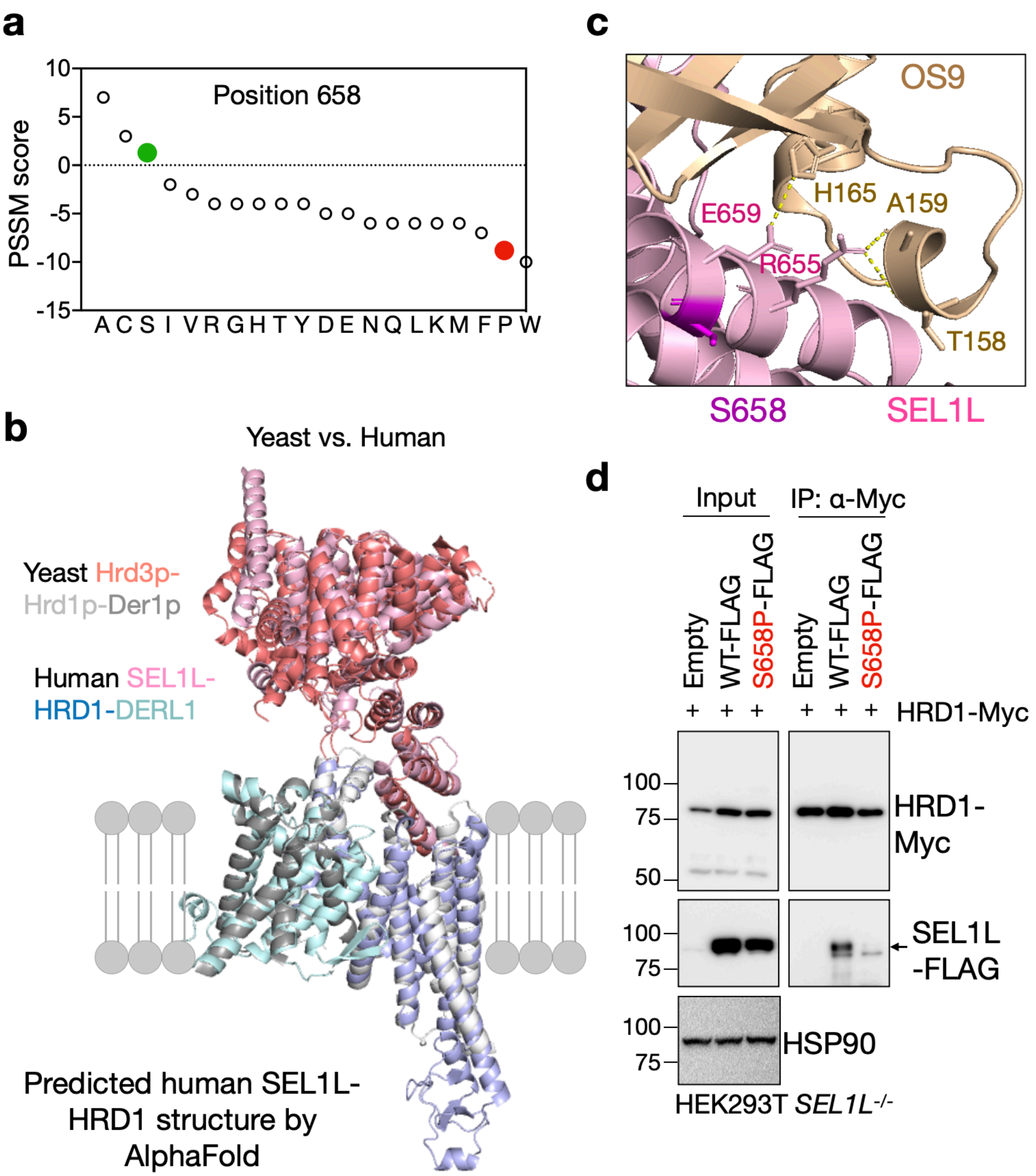

Supplementary Fig. 6. Sequence and structural analyses of SEL1L^{S658P} and the SEL1L-HRD1 complex.
(a) Position-specific scoring matrix (PSSM) score of various amino acids at SEL1L 658 position, with S658 in green and P658 in red. **(b)** Structure alignment of the human and yeast SEL1L-HRD1 complexed. **(c)** Side view of the SEL1L-OS9 interface around the SEL1L S658 position, with the dotted lines indicating SEL1L-OS9 interaction residues. **(d)** Immunoprecipitation of Myc-agarose in *SEL1L*$^{-/-}$ HEK293T cells transfected with indicated SEL1L-FLAG and HRD1-Myc constructs to examine the interaction between HRD1 and SEL1L (two independent repeats).

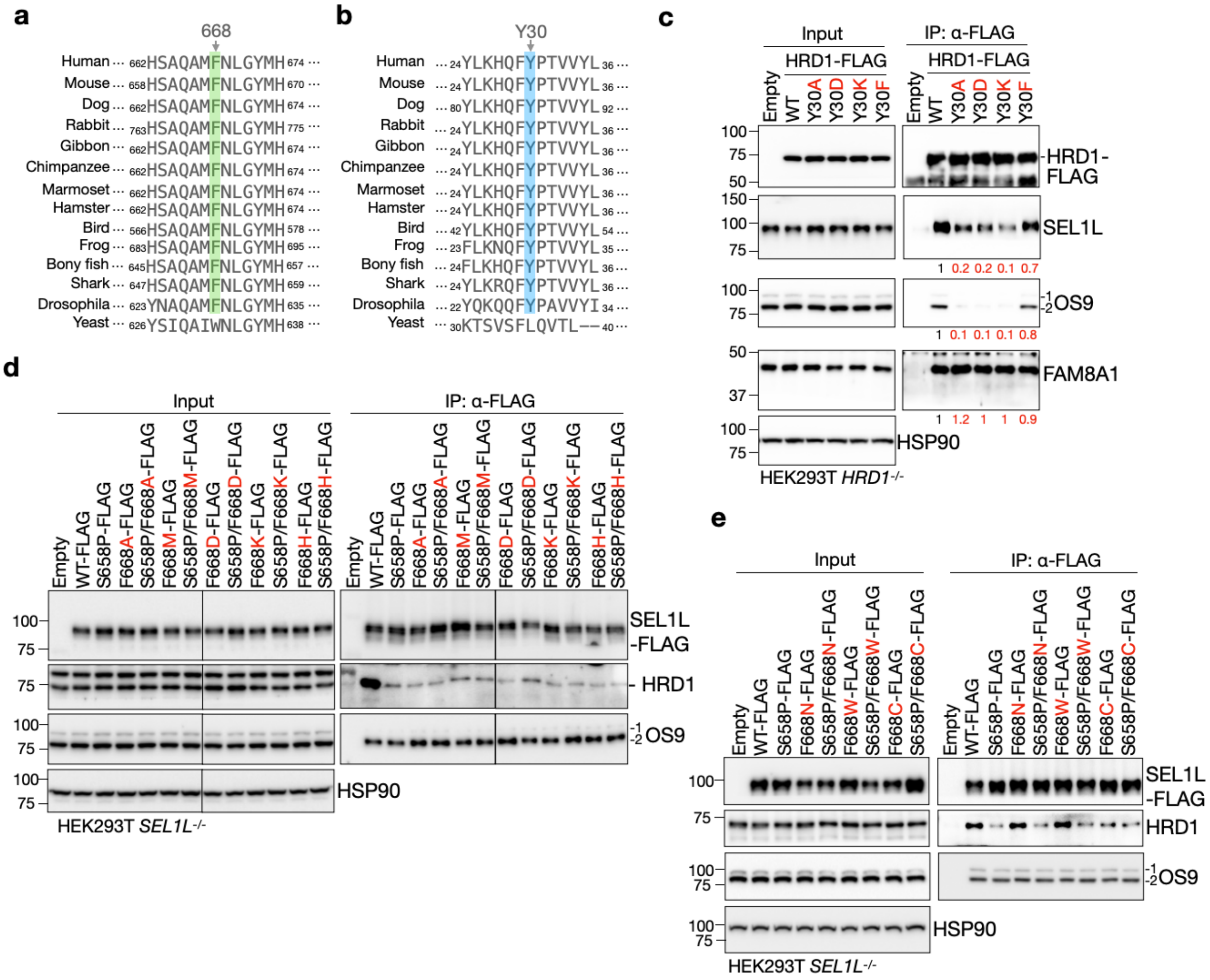

Supplementary Fig. 7. Biochemical analyses of HRD1-Y30 and SEL1L-F668.
(a-b) Amino acid sequence alignments of SEL1L and HRD1 showing the conservation of SEL1L-F668 (**a**, highlighted in green) and HRD1-Y30 (**b**, highlighted in blue) residues across species. **(c)** Immunoprecipitation of FLAG-agarose in *HRD1*$^{-/-}$ HEK293T cells expressing indicated HRD1-FLAG variants to exam their interaction with SEL1L, OS9, and HRD1's cofactor FAM8A1, with quantitation shown below the blots as mean values from two independent repeats. **(d-e)** Immunoprecipitation of FLAG-agarose in *SEL1L*$^{-/-}$ HEK293T cells expressing indicated SEL1L-FLAG variants to examine their interaction with HRD1 and OS9 (two independent repeats).

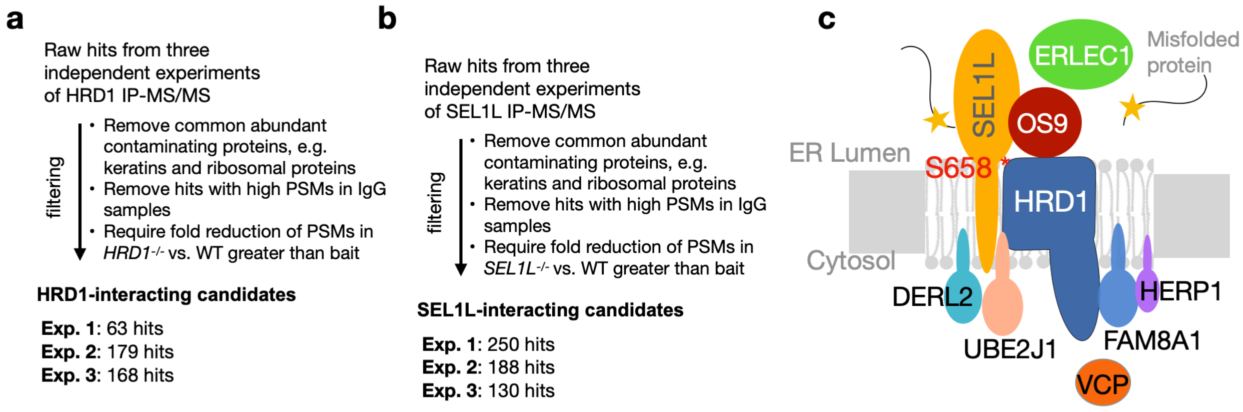

Supplementary Fig. 8. The validation and filtering criteria of the IP-MS experiments. **(a-b)** The flow of the HRD1- (**a**) and SEL1L- (**b**) IP-MS strategy to screen for HRD1/SEL1L-interacting proteins. **(c)** Diagram showing the protein components of the SEL1L-HRD1 ERAD complex.

Protein name	Uniprot ID	Repeat 1				Repeat 2 and Repeat 3					
		IgG	*HRD1$^{-/-}$*	*SEL1L$^{-/-}$*	WT	IgG Rep2/3	*HRD1$^{-/-}$* Rep2/3	*SEL1L$^{-/-}$* Rep2	WT Rep2	*SEL1L$^{-/-}$* Rep3	WT Rep3
SYVN1	Q86TM6	0	33	78	94	0	47	112	135	108	109
FAM8A1	Q9UBU6	0	19	49	62	0	8	57	70	48	66
ERLEC1	Q96DZ1	0	18	6	54	0	0	0	66	0	45
SEL1L	Q9UBV2	0	17	3	49	0	0	0	65	0	62
ABCD3	P28288	0	3	21	28	1	0	18	28	24	24
VDAC2	P45880	0	4	17	23	0	4	22	23	26	21
VCP	P55072	0	0	21	13	2	2	17	20	21	12
OS9	Q13438	0	5	1	25	0	0	0	53	0	36
VDAC3	Q9Y277	0	0	9	13	0	0	8	15	8	13
HACD3	Q9P035	0	0	3	7	0	0	1	2	1	2
HERPUD1	Q15011	0	0	7	2	0	1	4	5	6	2
KHDC1	Q4VXA5	0	1	1	6	0	0	2	9	1	7
SURF4	O15260	0	1	3	3	0	0	4	5	3	6
VDAC1	P21796	0	0	2	4	0	0	1	5	4	7
UBE2D3	P61077	0	0	0	4	0	0	0	4	0	7
DERL2	Q9GZP9	0	0	0	3	0	0	1	8	1	6
PRADC1	Q9BSG0	0	0	0	2	0	0	0	1	0	2
HM13	Q8TCT9	0	0	0	2	0	0	0	2	0	3
OMA1	Q96E52	0	0	0	2	0	0	0	3	1	1
CHERP	Q8IWX8	0	0	1	1	0	0	1	2	1	2
UNC50	Q53HI1	0	0	0	2	0	0	0	2	0	2
FABP5	Q01469	0	0	1	1	0	0	1	1	4	14
UBE2D1	P51668	0	0	0	2	0	0	0	2	0	2
TMUB1	Q9BVT8	0	0	0	1	0	0	2	1	1	2
UBE2J1	Q9Y385	0	0	0	0	0	0	0	1	0	2

Supplementary Table 1: HRD1-IP mass spectrometry result. 24 hits for HRD1 interacting proteins from three independent repeats. (UBE2J1 was detected in only two of three repeats; IgG_Rep2/3 and *HRD1$^{-/-}$*_Rpe2/3 served as negative controls for Repeat 2 and Repeat 3). The values in the table represent the total number of the sequenced peptides (PSMs value).

Protein name	Uniprot ID	Repeat 1				Repeat 2				Repeat 3			
		IgG	*SEL1L*$^{-/-}$	WT	*HRD1*$^{-/-}$	IgG	*SEL1L*$^{-/-}$	WT	*HRD1*$^{-/-}$	IgG	*SEL1L*$^{-/-}$	WT	*HRD1*$^{-/-}$
SEL1L	Q9UBV2	0	4	260	378	0	1	341	501	0	2	304	475
OS9	Q13438	0	0	199	386	0	0	241	301	0	0	190	340
ERLEC1	Q96DZ1	0	0	184	196	0	0	217	170	0	0	158	159
DPM1	O60762	0	3	27	26	0	1	19	10	0	0	24	22
FAM8A1	Q9UBU6	0	0	37	8	0	0	41	9	0	0	34	6
SYVN1	Q86TM6	0	0	31	14	0	0	27	6	0	0	23	11
UBE2J1	Q9Y385	0	0	10	31	0	0	14	24	0	0	11	30
SERPINH1	P50454	0	2	24	12	0	0	46	9	0	0	39	9
CANX	P27824	0	1	5	24	0	0	12	14	0	0	7	25
LRIG2	O94898	0	0	3	22	0	0	4	13	0	0	7	18
ATP1A1	P05023	0	1	5	17	0	0	8	10	0	1	8	9
RPN1	P04843	0	1	8	13	0	0	12	10	0	1	13	16
FBXO9	Q9UK97	0	0	9	12	0	0	6	4	0	1	11	2
PIGT	Q969N2	0	1	9	10	0	0	7	6	0	0	6	6
HYOU1	Q9Y4L1	0	0	5	13	0	0	5	7	0	0	4	11
PIGK	Q92643	0	0	2	15	0	0	3	9	0	0	3	12
TMEM201	Q5SNT2	0	0	13	4	0	0	13	1	0	0	12	8
SMIM14	Q96QK8	0	0	3	13	0	0	6	6	0	0	3	12
VCP	P55072	2	0	11	3	1	1	27	2	2	0	9	3
HM13	Q8TCT9	0	0	11	4	0	0	11	3	0	0	10	2
BSG	P35613	0	1	6	7	0	1	7	4	0	0	9	4
FOXRED2	Q8IWF2	0	0	2	12	0	0	12	9	0	0	8	15
PLOD2	O00469	0	0	6	7	0	0	2	0	0	0	2	1
DDOST	P39656	0	0	2	11	0	0	1	7	0	0	1	7
PRADC1	Q9BSG0	0	0	1	12	0	0	5	7	0	0	3	8
STT3A	P46977	0	0	2	8	0	0	3	12	0	0	1	6
TMEM33	P57088	0	0	1	8	0	0	2	4	0	0	2	3
DNAJC7	Q99615	0	0	4	5	0	0	3	2	0	0	11	11
PTK7	Q13308	0	0	1	8	0	0	1	2	0	0	2	3
NUP93	Q8N1F7	0	0	3	4	0	0	1	3	0	0	2	6
NCLN	Q969V3	0	0	1	6	0	0	3	1	0	0	3	3
LAMC1	P11047	0	0	2	4	0	0	2	0	0	0	2	3
LOX	P28300	0	0	2	4	0	0	1	2	0	0	1	2
YME1L1	Q96TA2	0	0	3	2	0	0	1	4	0	0	2	4
CHST12	Q9NRB3	0	0	3	2	0	0	1	3	0	0	4	5
GARS1	P41250	0	0	1	3	0	0	1	1	0	0	1	1
EDEM3	Q9BZQ6	0	0	4	0	0	0	4	0	0	0	2	1
EMILIN3	Q9NT22	0	0	4	0	0	0	2	0	0	0	3	1

TMEM9	Q9P0T7	0	0	4	0	0	0	2	1	0	0	3	3
HPSE	Q9Y251	0	0	3	0	0	0	8	0	0	0	3	1
VDAC3	Q9Y277	0	0	1	2	0	0	5	0	0	0	5	0
PIGS	Q96S52	0	0	1	2	0	0	3	3	0	0	2	1
JAM3	Q9BX67	0	0	2	0	0	0	3	0	0	0	3	0
CERS2	Q96G23	0	0	2	0	0	0	1	1	0	0	1	1
TMED9	Q9BVK6	0	0	1	1	0	0	3	2	0	0	2	2
TMTC3	Q6ZXV5	0	0	2	0	0	0	1	0	0	0	1	0
HERPUD1	Q15011	0	0	1	0	0	0	1	0	0	0	1	0

Supplementary Table 2: SEL1L-IP mass spectrometry result. 47 hits for SEL1L interacting proteins from three independent repeats. The values in the table represent the total number of the sequenced peptides (PSMs value).
