# Supplementary material for: SEL1L-HRD1 interaction is required to form a functional HRD1 ERAD complex
Source: Nat Commun. 2024 Feb 16;15:1440. doi: 10.1038/s41467-024-45633-0 (PMC10873344; doi:10.1038/s41467-024-45633-0)
Supplement: Supplementary file 3 — Description of Additional Supplementary Files [file 41467_2024_45633_MOESM3_ESM.pdf]

Description of Additional Supplementary Files

File name: Supplementary Movie 1

Description: Early-onset ataxia in *SEL1L*^{S658P} KI mice. Balance beam test of 6-week-old WT and KI mice. Animals were trained for two days before recording the test. Loss of coordination and balance in KI mice is shown at a regular speed and in a fourfold slowed motion.
