# Supplementary material for: SEL1L-HRD1 interaction is required to form a functional HRD1 ERAD complex
Source: Nat Commun. 2024 Feb 16;15:1440. doi: 10.1038/s41467-024-45633-0 (PMC10873344; doi:10.1038/s41467-024-45633-0)
Supplement: Supplementary file 5 — Reporting Summary [file 41467_2024_45633_MOESM5_ESM.pdf]

nature portfolio

Corresponding author(s): Ling Qi

Last updated by author(s): 01/15/2024

Reporting Summary

Nature Portfolio wishes to improve the reproducibility of the work that we publish. This form provides structure for consistency and transparency in reporting. For further information on Nature Portfolio policies, see our Editorial Policies and the Editorial Policy Checklist.

Statistics

For all statistical analyses, confirm that the following items are present in the figure legend, table legend, main text, or Methods section.

n/a	Confirmed	
☐	☒	The exact sample size (*n*) for each experimental group/condition, given as a discrete number and unit of measurement
☐	☒	A statement on whether measurements were taken from distinct samples or whether the same sample was measured repeatedly
☐	☒	The statistical test(s) used AND whether they are one- or two-sided<br>*Only common tests should be described solely by name; describe more complex techniques in the Methods section.*
☒	☐	A description of all covariates tested
☐	☒	A description of any assumptions or corrections, such as tests of normality and adjustment for multiple comparisons
☐	☒	A full description of the statistical parameters including central tendency (e.g. means) or other basic estimates (e.g. regression coefficient) AND variation (e.g. standard deviation) or associated estimates of uncertainty (e.g. confidence intervals)
☐	☒	For null hypothesis testing, the test statistic (e.g. *F*, *t*, *r*) with confidence intervals, effect sizes, degrees of freedom and *P* value noted<br>*Give P values as exact values whenever suitable.*
☒	☐	For Bayesian analysis, information on the choice of priors and Markov chain Monte Carlo settings
☒	☐	For hierarchical and complex designs, identification of the appropriate level for tests and full reporting of outcomes
☒	☐	Estimates of effect sizes (e.g. Cohen's *d*, Pearson's *r*), indicating how they were calculated

Our web collection on statistics for biologists contains articles on many of the points above.

Software and code

Policy information about availability of computer code

Data collection

For the SEL1L S658P KI mice generation, computer algorithm (http://crispor.tefor.net) was used for the two single guide RNAs (sgRNAs) design;

The individual structure models of human SEL1L, HRD1, OS9, and DERLIN1 were downloaded from AlphaFold2 database (https://alphafold.ebi.ac.uk/); The CryoEM structure of the yeast Hrd3p-Hrd1p-Der1p protein complex was downloaded from PDB ID: 6VJZ and Hrd3p-Yos9 complex was download from PDB ID: 6VK3; All the structure images of human SEL1L 171-723 aa, HRD1 1-334 aa, OS9 33-655 aa and DERLIN1 1-213 aa were rendered by PyMOL (version 2.3.2).

The amino acid sequence of human SEL1L (accession no. NP_005056.3) was aligned with the SEL1L homologs from chimpanzee (JAA44458.1), gibbon (XP_003260889.1), marmoset (XP_002754215.1), dog (XP_038530327.1), rabbit (XP_002719662.2), hamster (NP_001268291.1), mouse (NP_001034178.1), bird (XP_021143193.1), frog (XP_041430335.1), bony fish (NP_001038629.1), shark (XP_048393621.1), drosophila (NP_001262882.1) and yeast (QHB10358.1) by using ClustalW program.

The amino acid sequence of human HRD1 (SYVN1) (accession no. NP_115807.1) was aligned with the HRD1 homologs from chimpanzee (JAA39943.1), gibbon (XP_032009276.1), marmoset (JAB46248.1), dog (XP_038280973.1), hamster (XP_051049706.1), mouse (AAH46829.1), bird (XP_053824008.1), frog (XP_012816159.1), bony fish (AAH44465.1), shark (XP_043538543.1), drosophila (NP_001263152.1) and yeast (QHB11597.1) by using ClustalW program.

MRI acquisitions were performed on a 7T simultaneous PET-MR scanner (MR Solutions Ltd.). The 2D sagittal T2-weighted images (T2WI) were performed using fast spin echo (FSE) sequence (TR/TE: 4000/45 ms, echo train length of 7, eight average, 28 slices, slice thickness of 500 µm, in-plane resolution 390×140 µm3), to manually draw the regions of interests (ROIs) of cerebellum and cortex. The 3D T2Wis were performed

using Fast Low Angle Shot (FLASH) sequence (TR/TE: 50/5 ms, FA=30°, seven average, with an isotropic voxel resolution of 93×93×93 μm3) for 3D brain render reconstruction For the reconstruction, 3D images were performed using our in-house MATLAB code;
Western blot band intensity was determined using Image lab (Bio-Rad) software;
TEM images were collected with the use of JEM-1400 TEM.

For Mass Spectrometry, proteins were identified by searching the MS/MS data against UniProt entries using Proteome Discoverer (v2.4, Thermo Scientific). Search parameters included MS1 mass tolerance of 10 ppm and fragment tolerance of 0.2 Da; two missed cleavages were allowed; carbamidomethylation of cysteine was considered fixed modification and oxidation of methionine, deamidation of asparagine and glutamine were considered as potential modifications. False discovery rate (FDR) was determined using Percolator and proteins/peptides with an FDR of ≤1% were retained for further analysis.

Data analysis

All the structure images of human SEL1L residues 171-723, HRD1 1-334, OS9 33-655 and DERLIN1 1-213 were rendered by PyMOL (version 2.3.2);
To analyze the evolutionary conservation of the residue, a position- specific scoring matrix (PSSM) was generated from a PSI-BLAST search of the target protein through the NCBI NR database;
Sequence alignments were generated by ClustalW program;
The intensity inhomogeneity of images was corrected by N4 algorithm and then co-registered to the Mouse Magnetic Resonance Microscopy Atlas (https://www.loni.usc.edu/research/atlas_downloads). The masks of whole brain, cerebellum and cortex were segmented and manually edited using the ITK- SNAP software. The rendered 3D brain was reconstructed, and masks of cerebellum and cortex were displayed using the ParaView software version 5.10.1;
Transmission electron microscopy (TEM) images were taken by JEOL 1400-plus electron microscope (JEOL);
The target sites for CRISPR/Cas9 were selected using the web program (http://www.e-crisp.org/E-CRISP/designcrispr.html);
Statistics tests were performed in GraphPad Prism version 8.0 (GraphPad Software);
Immunofluorescence images were analyzed using the Fiji 2.0.0 software (Image J)

For manuscripts utilizing custom algorithms or software that are central to the research but not yet described in published literature, software must be made available to editors and reviewers. We strongly encourage code deposition in a community repository (e.g. GitHub). See the Nature Portfolio guidelines for submitting code & software for further information.

Data

Policy information about availability of data

All manuscripts must include a data availability statement. This statement should provide the following information, where applicable:

- Accession codes, unique identifiers, or web links for publicly available datasets
- A description of any restrictions on data availability
- For clinical datasets or third party data, please ensure that the statement adheres to our policy

Proteomics datasets for the HRD1 IP-MS and SEL1L IP-MS in HEK293T cells are available via ProteomeXchange with identifiers PXD043674 and PXD041882, respectively. The materials and reagents used are either commercially available or available upon request. All other data are available in the main text or in the supplementary information and Source Data Files. Source data are provided with the paper.

Research involving human participants, their data, or biological material

Policy information about studies with human participants or human data. See also policy information about sex, gender (identity/presentation), and sexual orientation and race, ethnicity and racism.

Reporting on sex and gender	N/A
Reporting on race, ethnicity, or other socially relevant groupings	N/A
Population characteristics	N/A
Recruitment	N/A
Ethics oversight	N/A

Note that full information on the approval of the study protocol must also be provided in the manuscript.

Field-specific reporting

Please select the one below that is the best fit for your research. If you are not sure, read the appropriate sections before making your selection.

☒ Life sciences ☐ Behavioural & social sciences ☐ Ecological, evolutionary & environmental sciences

For a reference copy of the document with all sections, see nature.com/documents/nr-reporting-summary-flat.pdf

Life sciences study design

All studies must disclose on these points even when the disclosure is negative.

Sample size	Based on sample size formula of the power analysis, N=8(CV)^2[1+(1-PC)^2]/(PC)^2, to reach the error = 0.05, Power = 0.80, percentage change in means (PC) = 20%, co-efficient of variation (CV) = 10 ~ 15% (varies between the experiments).<br>The sample size for each animal experiment was described in the figure legend.
Data exclusions	No animals or samples were excluded from the analysis.
Replication	All experiments were repeated at least twice or performed with independent samples. All experiments were successfully repeated. This is described in text methods part, section "Statistical Analysis". The exact repeat times of experiments are indicated in the figure legends.
Randomization	Mice were randomly assigned based on the age, genotype and gender. Cells were grown under the same conditions and randomly allocated into different groups without any bias.
Blinding	When experiments were done by one investigator, blindness is not applied or possible. When experiments are done sequentially by different investigators, investigators were blinded to allocation during experiments and outcome assessment.

Reporting for specific materials, systems and methods

We require information from authors about some types of materials, experimental systems and methods used in many studies. Here, indicate whether each material, system or method listed is relevant to your study. If you are not sure if a list item applies to your research, read the appropriate section before selecting a response.

Materials & experimental systems

n/a	Involved in the study	
☐	☒	Antibodies
☐	☒	Eukaryotic cell lines
☒	☐	Palaeontology and archaeology
☐	☒	Animals and other organisms
☒	☐	Clinical data
☒	☐	Dual use research of concern
☒	☐	Plants

Methods

n/a	Involved in the study	
☒	☐	ChIP-seq
☒	☐	Flow cytometry
☐	☒	MRI-based neuroimaging

Antibodies

Antibodies used	Antibodies used for Western Blot were:<br>anti-HSP90 (Santa Cruz, #sc-13119, 1:5,000), anti-SEL1L (home-made, 1:10,000) 44, anti-HRD1 (Proteintech, #13473-1, 1:2,000), anti-OS9 (Abcam, #ab109510, 1:5,000), anti-CD147 (Proteintech, #11989-1, 1:3,000), anti-IRE1α (Cell Signaling, #3294, 1:2,000), anti-ERLEC1 (Abcam, #ab181166, 1:5,000), anti-UBE2J1 (Santa Cruz, #sc-377002, 1:3,000), anti-DERL2 (gift from Chih-Chi Andrew Hu, 1:1,000), anti-BiP/GRP94 (Abcam, #ab21685, 1:5,000), anti-PDI (Enzo, #ADI-SPA-890, 1:5,000), anti-FLAG (Sigma, #F1804, 1:1,000), anti-HA (Sigma, #H3663, 1:5,000), anti-Myc (Sigma, #C3956, 1:3000), anti-Pro-Caspase-3 (Cell Signaling, #9662, 1:2,000), anti-cleaved-Caspase-3 (Cell Signaling, #9661, 1:1,000), anti-Calbindin (Cell Signaling, #2173, 1:5,000), anti-PERK (Cell Signaling, #3192, 1:5000), anti-p-PERK (Cell Signaling, #3179, 1:1000), anti-eIF2α (Cell Signaling, #9722, 1:5000), anti-p-eIF2α (Cell Signaling, #9721, 1:1000), anti-VCP (Proteintech, #10736-1-AP, 1:3000), anti-HERP1 (Abcam, #ab150424, 1:3000), anti-FAM8A1 (Proteintech, #24746-1-AP,1:3000), anti-CHOP (Cell Signaling, #2895S, 1:1000), anti-GFAP (Cell Signaling, #3670S, 1:3000), anti-IbaI (Proteintech, #10904-1, 1:3000).<br><br>Secondary antibody: anti-Rabbit IgG HRP conjugate (Bio-Rad, #1706515, 1:10,000), anti-Mouse IgG HRP conjugate (Bio-Rad, #1706516, 1:10,000), anti-Rabbit IgG TrueBlot HRP (Rockland, #18-8816-33, 1:500), anti-Mouse IgG TrueBlot-HRP (Rockland, #18-8817-31, 1:500).<br><br>Antibodies used for immunoprecipitation were:<br>Anti-SEL1L (home-made), anti-HRD1 (home-made), anti-HRD1 (Cell Signaling, #14773S).<br>Antibodies used for immunofluorescence were:<br>anti-Calbindin (Cell Signaling, #2173, 1:100); anti-KDEL (Novus Biologicals, #97469, 1:200); anti-NeuN (Sigma, #ABN90, 1:200); anti-GFAP (Cell Signaling, #3670, 1:100); anti-Iba1 (Fujifilm, #019-19741, 1:100).
Validation	Home-made antibodies: anti-SEL1L: validated for Western blot and immunofluorescence (Zhou et al., Science 2020) in mouse and human. We validated the application in immunoprecipitation in HEK293T cells and mouse tissues in this study (Figure 6 and 8).<br>anti-HRD1: We validated the application in Western blot and immunoprecipitation in HEK293T cells and mouse tissues in this study (Figure 6-9). anti-DERL2 (gift from Chih-Chi Andrew Hu): validated for Western blot in human and mouse in (Dougan et al., Molecular and Cellular Biology) and (Kriss et al., Blood, 2012).<br><br>Commercial antibodies were validated by manufactures.<br>Anti-HSP90 (Santa Cruz, #sc-13119): validated for Western blot in human and mouse (https://www.scbt.com/p/hsp-90alpha-beta-

antibody-f-8);
anti-HRD1 (Proteintech, #13473-1): validated for Western blot in human and mouse (https://www.ptglab.com/products/SYVN1-Antibody-13473-1-AP.htm);
anti-OS9 (Abcam, #ab109510): validated for Western blot in human and mouse (https://www.abcam.com/os9-Antibody-epr42722-ab109510.html);
anti-CD147 (Proteintech, #11989-1): validated for Western blot in human and mouse (https://www.ptglab.com/products/BSG-Antibody-11989-1-AP.htm);
anti-IRE1α (Cell Signaling, #3294): validated for Western blot in human and mouse (https://www.cellsignal.com/products/primary-antibodies/ire1a-14c10-rabbit-mab/3294);
anti-ERLEC1 (Abcam, #ab181166): validated for Western blot in human and mouse (https://www.abcam.com/products/primary-antibodies/erlec1-antibody-epr13849-ab181166.html);
anti-UBE2J1 (Santa Cruz, #sc-377002): validated for Western blot in human and mouse (https://www.scbt.com/p/ube2j1-antibody-b-6);
anti-BiP/GRP94 (Abcam, #ab21685): validated for Western blot in human and mouse (https://www.abcam.com/products/primary-antibodies/grp78-bip-antibody-ab21685.html).
anti-PDI (Enzo, #ADI-SPA-890): validated for Western blot in human and mouse (https://www.enzolifesciences.com/ADI-SPA-890/pdi-polyclonal-antibody/);
anti-FLAG (Sigma, #F1804): validated for Western blot in human and mouse (https://www.sigmaaldrich.com/US/en/product/sigma/f1804);
anti-HA (Sigma, #H3663): validated for Western blot in human and mouse (https://www.sigmaaldrich.com/US/en/product/sigma/h3663?=kr);
anti-Myc (Sigma, #C3956): validated for Western blot in human and mouse (https://www.sigmaaldrich.com/US/en/product/sigma/c3956);
anti-Pro-Caspase-3 (Cell Signaling, #9662): validated for Western blot in human and mouse (https://www.cellsignal.com/products/primary-antibodies/caspase-3-antibody/9662);
anti-cleaved-Caspase-3 (Cell Signaling, #9661): validated for Western blot in human and mouse (https://www.cellsignal.com/products/primary-antibodies/cleaved-caspase-3-asp175-antibody/9661);
anti-Calbindin (Cell Signaling, #2173): validated for Western blot and immunofluorescence in mouse (https://www.cellsignal.com/products/primary-antibodies/calbindin-c26d12-rabbit-mab/2173) and we validated for immunofluorescence in this study (Extended Data Figure 2).
anti-PERK (Cell Signaling, #3192): validated for Western blot in human and mouse (https://www.cellsignal.com/products/primary-antibodies/perk-c33e10-rabbit-mab/3192);
anti-p-PERK (Cell Signaling, #3179: validated for Western blot in human and mouse (https://www.cellsignal.com/products/primary-antibodies/phospho-perk-thr980-16f8-rabbit-mab/3179);
anti-eIF2α (Cell Signaling, #9722): validated for Western blot in human and mouse (https://www.cellsignal.com/products/primary-antibodies/eif2a-antibody/9722);
anti-p-eIF2α (Cell Signaling, #9721): validated for Western blot in human and mouse (https://www.cellsignal.com/products/primary-antibodies/phospho-eif2a-ser51-antibody/9721);
anti-VCP (Proteintech, #10736-1-AP): validated for Western blot in human and mouse (https://www.ptglab.com/products/VCP-Antibody-10736-1-AP.htm)
anti-HERP1 (Abcam, #ab150424): validated for Western blot in human (https://www.abcam.com/products/primary-antibodies/herpud1-antibody-epr9649-ab150424.html);
anti-FAM8A1 (Proteintech, #24746-1-AP): validated for Western blot in human (https://www.ptglab.com/products/FAM8A1-Antibody-24746-1-AP.htm);
anti-CHOP (Cell Signaling, #2895S): validated for Western blot in human and mouse (https://www.cellsignal.com/products/primary-antibodies/chop-l63f7-mouse-mab/2895);
anti-GFAP (Cell Signaling, #3670S): validate for Western blot and immunofluorescence in mouse (https://www.cellsignal.com/products/primary-antibodies/gfap-ga5-mouse-mab/3670);
anti-Ibal (Proteintech, #10904-1): validate for Western blot and immunofluorescence in mouse (https://www.ptglab.com/products/IBA1-Antibody-10904-1-AP.htm);
anti-HRD1 (Cell Signaling, #14773S): validate for Western blot and immuoprecipitation in human (https://www.cellsignal.com/products/primary-antibodies/syvn1-d3o2a-rabbit-mab/14773);
anti-KDEL (Novus Biologicals, #97469): validate for immunofluorescence in human and mouse (https://www.novusbio.com/products/kdel-antibody-10c3_nbp1-97469);
anti-NeuN (Sigma, #ABN90): validate for immunofluorescence in mouse (https://www.sigmaaldrich.com/US/en/product/mm/abn90);
anti-Iba1 (Fujifilm, #019-19741): validate for immunofluorescence in mouse (https://www.fujifilmcdi.com/anti-iba1-polyclonal-antibody-019-19741).

Eukaryotic cell lines

Policy information about cell lines and Sex and Gender in Research

Cell line source(s)	HEK293T cells were originally obtained from ATCC. The information is described in the Methods.
Authentication	The cells have been authenticated by morphology.
Mycoplasma contamination	No Mycoplasma contamination after testing.
Commonly misidentified lines (See ICLAC register)	The cell lines are not listed in that database.

Animals and other research organisms

Policy information about studies involving animals; ARRIVE guidelines recommended for reporting animal research, and Sex and Gender in Research

Laboratory animals	Species: Mus musculus;<br>Sex: Female/Male; Age: 2-48 weeks; Information of sex and age was indicated in the figures and figure legends;<br>Strain: All mice were in C57BL/6J and SJL/J mixed background.<br>All mice were housed in a pathogen-free animal facility at 22 ± 1°C on a 12-hr light/dark cycle with 40-60% humidity and fed a low-fat diet (13% fat, 57% carbohydrate, and 30% protein, LabDiet 5LOD), unless otherwise indicated.
Wild animals	No wild animals was used in this study.
Reporting on sex	Both sexes of mice were used in this study.
Field-collected samples	No field-collected samples was used in this study.
Ethics oversight	All animal procedures were approved by the Institutional Animal Care and Use Committee of the University of Michigan Medical School (PRO0008989 and PRO00010658) and University of Virginia (4459-08-23) in accordance with the National Institutes of Health (NIH) guidelines.

Note that full information on the approval of the study protocol must also be provided in the manuscript.

Magnetic resonance imaging

Experimental design

Design type	Structural mouse brain imaging
Design specifications	Anesthetized mice were transcardially perfused with 4% paraformaldehyde in PBS. The head were cut and removed the skin and soft tissue and then soaked in PBS containing 5% Gd-DTPA and stored in 4°C for 5 days. Prior to imaging, the brain specimens were placed in a 15 ml tube containing proton signal-free susceptibility-matched fluid (Galden Heat Transfer Fluid, HT230. SOLVEY, Italy), which was placed in a mouse head coil. MRI acquisitions were performed on a 7T simultaneous PET-MR scanner (MR Solutions Ltd.) at the Zilkha Neurogenetic Institute (University of Southern California). The 2D sagittal T2-weighted images (T2WI) were performed using fast spin echo (FSE) sequence (TR/TE: 4000/45 ms, echo train length of 7, eight average, 28 slices, slice thickness of 500 µm, in-plane resolution 390×140 µm3), to manually draw the regions of interests (ROIs) of cerebellum and cortex. The 3D T2Wis were performed using Fast Low Angle Shot (FLASH) sequence (TR/TE: 50/5 ms, FA=30°, seven average, with an isotropic voxel resolution of 93×93×93 µm3) for 3D brain render reconstruction.
Behavioral performance measures	Structural mouse brain imaging was performed without behavioral task.

Acquisition

Imaging type(s)	structural imaging
Field strength	1T and 3T
Sequence & imaging parameters	The 2D sagittal T2-weighted images (T2WI) were performed using fast spin echo (FSE) sequence (TR/TE: 4000/45 ms, echo train length of 7, eight average, 28 slices, slice thickness of 500 µm, in-plane resolution 390×140 µm3), to manually draw the regions of interests (ROIs) of cerebellum and cortex. The 3D T2Wis were performed using Fast Low Angle Shot (FLASH) sequence (TR/TE: 50/5 ms, FA=30°, seven average, with an isotropic voxel resolution of 93×93×93 µm3) for 3D brain render reconstruction.
Area of acquisition	Whole brain

Diffusion MRI ☐ Used ☒ Not used

Preprocessing

Preprocessing software	For the reconstruction, 3D images were performed using our in-house MATLAB code. The intensity inhomogeneity of images was corrected by N4 algorithm and then co-registered to the Mouse Magnetic Resonance Microscopy Atlas (https://www.loni.usc.edu/research/atlas_downloads). The masks of whole brain, cerebellum and cortex were segmented and manually edited using the ITK-SNAP software. The rendered 3D brain was reconstructed, and masks of cerebellum and cortex were displayed using the ParaView software version 5.10.1.
Normalization	ParaView software default pipeline

Normalization template	ParaView software default pipeline
Noise and artifact removal	ParaView software default pipeline
Volume censoring	ParaView software default pipeline

Statistical modeling & inference

Model type and settings	Structural MRI data was analyzed without modeling
Effect(s) tested	The error measures were tested for statistical significance by two-tailed Student's t-test.

Specify type of analysis: ☐ Whole brain ☐ ROI-based ☒ Both

Anatomical location(s): cerebellum and cortex

Statistic type for inference (See Eklund et al. 2016)	two-tailed Student's t-test was used and statistic comparisons are reported with exact p values.
Correction	No corrections in the main analysis

Models & analysis

n/a	Involved in the study
☒	☐ Functional and/or effective connectivity
☒	☐ Graph analysis
☒	☐ Multivariate modeling or predictive analysis
